# Clinical Activity and Tolerability of SL-401 (Tagraxofusp): Recombinant Diphtheria Toxin and Interleukin-3 in Hematologic Malignancies

**DOI:** 10.3390/biomedicines7010006

**Published:** 2019-01-05

**Authors:** Omar Alkharabsheh, Arthur E. Frankel

**Affiliations:** Division of Medical Oncology, Mitchell Cancer Institute, University of South Alabama, Mobile, AL 36604, USA; afrankel@health.southalabama.edu

**Keywords:** SL-401 (tagraxofusp), diphtheria immunotoxin, adverse events, Myeloid neoplasms

## Abstract

Overcoming the leukemia stem cell resistance to intensive chemotherapy has been an area of extensive research over the last two decades. Advances and greater understanding of the molecular biology of leukemia stem cells are in rapid progress. Targeted therapies are currently being used in clinical practice with reasonable response rates, but a cure is being achieved in only a small percentage of patients, most likely due to tumor mutational heterogeneity. A genetically engineered diphtheria toxin fused with interleukin-3 (SL-401 or tagraxofusp) has shown robust activity in blastic plasmacytoid dendritic cell neoplasm and promising response rates in different myeloid malignancies, including eradication of minimal residual disease. Multiple clinical trials are being conducted using this drug and the preliminary results are encouraging. This article reviews the clinical trials for SL-401, its mechanism of action, clinical activity, and the adverse event profile.

## 1. Introduction

The current treatment approach for myeloid malignancies is heavily dependent on intensive chemotherapy regimens that follow the old strategy of “one size fits all”. Novel targeted agents are emerging in clinical practice and clinical trials. Data on their efficacy and tolerability are promising, although no treatment has produced a cure thus far [1]. Intensive chemotherapy treatments are designed to induce hypocellular marrow and allow hematopoietic stem cells to recover and resume normal production of blood cell components. The durability and duration of remission by this kind of therapy can vary and will depend primarily on the mutational signature of the disease and epigenetic aberration. In addition, the planned consolidation strategy, whether it is allogeneic stem cell transplantation or further consolidative chemotherapy, has an effect on leukemia-free survival and how deeply we can eradicate leukemic stem cells. New agents that target actionable mutations, such as FLT3 and IDH, have been approved for clinical practice [2,3]. However, they have not had a significant enough effect on overall survival rates to replace chemotherapy, partly because of the diversity of cytogenetic abnormalities and the clonal evolution of new mutations. Nevertheless, it is important to assess the performance status and comorbidities of these treatments before exploring which treatment approach is the most appropriate in terms of toxicity and survival. Unfortunately, even with the most intensive treatment, the risk of relapse is high in the first two years, especially in patients with adverse cytogenetic profiles [4]. Extensive time and effort were spent in clinical trials optimizing the sequence of well-known acute leukemia chemotherapy protocols. There is a need to revolutionize the treatment of acute leukemia.

Unlike traditional chemotherapy, the specificity of the target on the tumor cells is the focus of the new era in cancer treatment. Various examples have been investigated in clinical trials, and many agents have been approved and are available in clinical practice. These include, for example, tyrosine kinase inhibitors to target the FLT3 mutation, monoclonal antibodies, bispecific antibodies, and chimeric antigen receptor T cell therapies. The ideal target, for example, in acute myeloid and lymphoid leukemia, is highly expressed in leukemic blasts, with minimal or no expression on normal hematopoietic cells, to induce deeper remission. Targets that have been studied in acute myeloid leukemia include CD33, CD135, FLT3, CXCR4, and vascular endothelial growth factor [5]. This article focuses on the diphtheria toxin-interleukin 3 fusion protein as a targeted therapy to leukemic blasts.

Hematopoiesis is a complex process with multiple factors, undertaken to avoid the production of clones and leukemic stem cells. Interleukin 3 (IL-3) is one of the growth factors and cytokines that participate in this process at the level of granulocytic and monocytic lineage [6]. Expression of the interleukin 3 receptor (IL-3R) starts at the CD34^+^ hematopoietic cell and is maintained during all stages of development for granulocytes and monocyte precursors. Multiple investigators have targeted this receptor for the treatment of different types of myeloid leukemia [7]. Most recently, experiments have used chimeric antigen receptor T cell technology to target CD123 in Hodgkin lymphoma in an effort to overcome the immunosuppressed microenvironment and allow the patient’s T lymphocytes to attack tumor cells [8]. The structure of IL-3R consists of an α subunit, which is the site of ligand attachment and represents the specificity of the receptor, and a β subunit, which is shared with the granulocyte-macrophage colony-stimulating factor and is important for signal transduction, internalization of the ligand–receptor complexes, and activation through phosphorylation of the Ras pathway [9].

The development of monoclonal antibodies and cytokines was a major advance in cancer treatment because of their ability to deliver cancer therapies, such as cytotoxic drugs, isotopes, and toxins. Toxins are powerful pro-apoptosis agents, and they achieve their cellular effect by various pathways. One of them is by shutting down protein synthesis [10]. After removal of the natural binding domain of the toxins, they are conjugated to a monoclonal antibody or cytokine as their ligand [11]. One toxin that has been used in cancer therapy is the diphtheria toxin, which is an exotoxin secreted by *Corynebacterium diphtheriae* [12]. This toxin inhibits protein synthesis in the host, which is the underlying pathophysiology of diphtheria infection [8]. Previously, the diphtheria toxin was engineered to target interleukin-2 and was approved by the Food and Drug Administration (FDA) in 2002 for the treatment of cutaneous T cell lymphoma [13]. However, because of the side effects, marketing of this drug was discontinued by the manufacturer.

**Figure 1:**
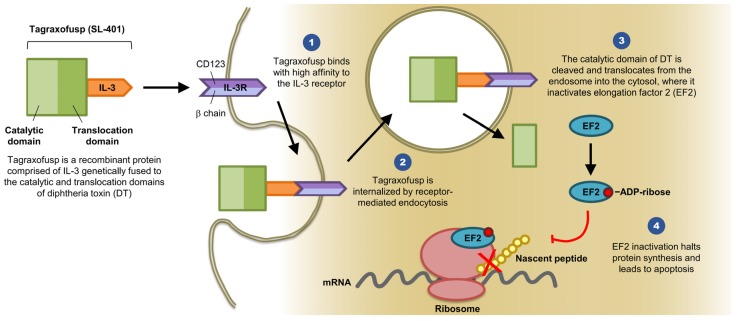
SL-401 Structure and Mechanism of Action (provided by Stemline Therapeutics, Inc., New York, NY 10022, USA). Abbreviations: IL-3, interleukin 3; IL-3R, interleukin 3 receptor; DT, diphtheria toxin; EF2, elongation factor 2; mRNA, messenger ribonucleic acid; ADP, adenosine diphosphate.

Diphtheria toxin consists of three domains and several linkers elements: The C terminus binding domain, the N terminus catalytic domain, and the translocation domain [14]. IL-3 is a cytokine that promotes the differentiation of hematopoietic stem cells into various myeloid cells [12]. IL-3R is composed of two subunits (α and β). The α subunit (CD123) directly binds to IL-3, whereas the β subunit (CDw131) functions as the signaling subunit (see Figure 1) [10]. The idea is to remove the diphtheria toxin binding domain and replace it with IL-3 to target leukemic stem cells that express CD123 and CDw131 by inhibiting intracellular protein synthesis and therefore inducing cell death.

This combination (diphtheria toxin and IL-3) is named SL-401 (tagraxofusp) and has reached phase II clinical trials, with robust activity in the blastic plasmacytoid dendritic cell neoplasm (BPDCN) and other hematological malignancies [15,16,17,18,19,20]. Subsequently, the FDA approved this drug in December 2018 for adult and pediatric BPDCN. In this review, we report the preclinical data and describe the clinical trials for SL-401 and its activity in various myeloid malignancies.

## 2. Preclinical Data and Experiments

Previously, investigators fused the truncated diphtheria toxin with GM-CSF using a His-Met linker. However, due to the toxicity profile and the hepatic side effects, they decided to explore a different growth factor [21]. The level of IL-3R expression was examined by Testa and colleagues and they demonstrated a higher level of IL-3R α chain in leukemic stem cells (LSC) and a low expression on normal hematopoietic stem cells, which makes it a marker for LSC and a target for treatment [2]. Using genetic engineering, the first 388 amino acid residues of the diphtheria toxin are fused with interleukin 3 via H-M linker (DT_388_IL3). The mechanism of action starts when the ligand is attached to the IL-3R α subunit (CD123). Along with binding to the IL-3R β subunit, this will trigger receptor-mediated endocytosis into a vesicle which will deliver the diphtheria toxin to the endosomes where it is cleaved by furin to generate the A and B fragments of the toxin. The acidic environment of the endosomes will partially unfold all three toxin domains to facilitate the escape of the A fragment that contains the catalytic domain into the cytosol. Finally, the catalytic domain will ADP-ribosylate the elongation factor 2, which will lead to protein synthesis inhibition and cell death [22] (see Figure 1). Additionally, several animal studies confirmed the in vivo efficacy of the engineered diphtheria toxin and provided more information about the appropriate dose and its readiness for the first human clinical trials [14,15]. To examine the level of expression on different myeloid lineage, researchers from Mayo Clinic performed a study of CD123 expression on myeloproliferative neoplasm subtypes with flow cytometry. They reported expression mostly on systemic mastocytosis, clonal eosinophilia, and a minor population of myelofibrosis [16]. This broadened the potential activity on several myeloid disorders, along with the previously described leukemic stem cells in acute myeloid leukemia. To understand more about the bone marrow microenvironment and its effect on disease pathogenesis, along with the possible use of this agent as an investigational drug in clinical trials, researchers at Dana-Farber Cancer Institute demonstrated that plasmacytoid dendritic cells (pDCs) play a role in protecting clonal plasma cells in multiple myeloma from treatment. Additionally, they reported that SL-401 induces pDC apoptosis, which resulted in overcoming treatment resistance in multiple myeloma [17].

## 3. Clinical Trials

The first in human trial was presented at the American Society of Clinical Oncology (ASCO) annual meeting in 2006. The preliminary findings showed manageable toxicity in elderly patients with acute myeloid leukemia (AML) or in those with relapsed or refractory disease [18]. The study included 31 patients who received one cycle of SL-401. The findings showed mild toxicity and promising biologic activity. At the 2007 ASCO annual meeting, investigators reported a patient with AML who achieved complete remission (CR) for eight months as well as two cases of partial response (PR) [19]. These patients were heavily pretreated, including bone marrow transplantation, and the cohort included patients with secondary AML either from a previous myelodysplastic syndrome or therapy-related AML. The major side effects reported in this abstract were mild to moderate and included transient fever, chills, hypotension, and hypoalbuminemia [19]. Table 1 highlights the clinical trials for SL-401, adverse events, and response rates.

Phase I data were first published in 2008 [20] and highlighted the following points. First, the maximum tolerated dose is 12.5 μg/kg/day, which can be infused safely over 15 min, twice daily every 48 h with a total of six doses as one cycle. Second, toxicity and side effects were manageable, and no grade IV/V adverse events were reported. Grade II/III adverse events included transaminitis, fever, hypoalbuminemia, hypotension, and hypocalcemia. This cohort included elderly patients, most of whom had a disease that was refractory to standard therapy. The median age was 67 years, and only 11% of patients had previously untreated AML. In addition, 96% of patients had an intermediate cytogenetic profile. The trial reported one case of CR and two cases of PR (Both AML and MDS). All of the patients who showed some form of response had relapsed or refractory disease and the patients with previously untreated AML showed no response to the experimental treatment [20].

The high level of IL-3R expression on BPDCN [23] provided the basis for investigation of SL-401 activity in this type of myeloid disease. Because BPDCN is an aggressive tumor, unfortunately, patients have a short survival, irrespective of the intensity of chemotherapy [24]. This accelerated the pathway to move SL-401 from in vitro studies to more early phase clinical trials [13,25]. The first prospective trial in BPDCN using SL-401 published its results and outcomes in 2014. Frankel et al. reported the detailed toxicity profile and response rates for the phase I/II trial [26]. The maximum tolerated dose was 12.5 μg/kg/day for five days in 11 patients with BPDCN. The reported adverse events were grade II/III, including edema, hypoalbuminemia, fever, chills, and transaminitis as well as one case of grade IV thrombocytopenia that was not related to an underlying thrombotic microangiopathy. Another adverse event that the investigators described in their outcomes data is vascular leak syndrome (VLS). This was reported as mild to moderate, and it manifested clinically with edema, with laboratory findings showing hypoalbuminemia. The VLS phenomenon had been reported in other fusion proteins with toxins [27]. Of the patients who underwent evaluation for response, 78% had an objective response, including 55% CR after the first cycle. Because of the nature of the drug, there is a question about whether the antibody developed because of previous diphtheria vaccination during childhood or whether the infused drug itself may trigger antibody development that might inactivate SL-401. The investigators examined this theory and reported that pretreatment titers were positive as a result of previous vaccination. A repeat evaluation after therapy showed an increased titer; however, this did not affect toxicity events, pharmacokinetics, or the clinical response rate [26].

The current advances in measuring minimal residual disease (MRD) have shown that conventional chemotherapy may not provide the deepest response or subsequently a better outcome. For example, the use of traditional consolidation chemotherapy after achieving CR in AML might not be enough to reduce the risk of relapse. For that, new treatment strategies are needed. Currently, a phase I/II study (NCT02270463) is underway in patients with AML who are at risk for relapse based on intermediate-high risk cytogenetics and are not candidates for allogeneic stem cell transplant. The following initial results were reported at the 2016 ASH annual meeting. First, the adverse event profile was similar to that of other clinical trials of SL-401, with a maximum tolerated dose of 12 μg/kg/day [28]. Second, the concept of targeting MRD-positive AML with SL-401 is promising, and the trial will continue to recruit patients until December 2018 (www.clinicaltrials.gov).

For multiple myeloma, studies are still in the early phase, with evidence showing that SL-401 targets myeloma stem cells [29]. As with pDC in the marrow microenvironment, those cells express CD123 and have a role in the proliferation of myeloma, which makes SL-401 a promising agent for myeloma backbone therapy. Currently, a phase I/II trial (NCT02661022) is recruiting patients with relapsed or refractory myeloma to be treated with pomalidomide, dexamethasone, and SL-401 [30].

The current evidence from early clinical trials indicates that side effects are tolerable and manageable. Additionally, clinical activity is seen in tumors that express CD123, especially BPDCN. Recently, investigators showed robust data at the American Society of Hematology annual meeting in December 2017 for a phase II trial of single-agent SL-401 in BPDCN, with an 84% overall response rate. Additionally, two abstracts were presented at the European Society of Hematology annual meeting in June 2018. The first one described chronic myelomonocytic leukemia, and all patients showed a spleen reduction response, while 12.5% showed marrow CR. The second abstract described primary myelofibrosis, and 50% of patients showed a spleen response. In summary, the results of trials of SL-401 indicate no serious adverse events, unlike other forms of immunotherapy, and a convening clinical activity in various hematologic malignancies, most effectively in BPDCN. In the future, we will likely see more positive results from phase III trials.

## 4. Side Effects and Tolerability

The currently reported side effects and clinical activity from early-phase trials are shown in Table 1. To review those in detail, this is a recombinant protein from diphtheria toxin conjugated with IL-3 to internalize the toxin inside the tumor cells. Based on the nature of this agent, some form of immune or infusion reaction would be expected. The most serious adverse event that was reported in human clinical trials was capillary leak syndrome (CLS), previously called VLS, which is manifested by edema, hypoalbuminemia, hypotension, and fatigue. This was reported as grade ≤3; however, three cases of fatal CLS were reported in the BPDCN trial (www.ashclinicalnews.org).

Other significant side effects were reported up to grade 4, including thrombocytopenia. However, it was reversible and was not associated with a major bleeding event. In addition, less than grade 3 neutropenia was reported. Patients with marrow-involved malignancies are susceptible to cytopenia because of disease expansion and the side effects of treatment. As shown in Table 1, the rest of the side effects were tolerable and reversible, so one could argue that these patients have refractory disease and are heavily pretreated, and this by itself can explain the cytopenia. Additionally, transaminitis has been reported across trials, but it was reversible and less than or equal to grade 3.

## 5. Pediatric Experience

Although BPDCN is a disease of the elderly, a few patients who were younger than 18 years old were included for compassionate use because their disease was refractory to standard therapy. Sun et al. from the City of Hope reported the first three cases of pediatric patients with this type of malignancy. Overall, the treatment was very well tolerated, with side effects similar to those of CLS and infusion reaction [31]. The number of patients in this report was very small, so a conclusion about the efficacy of this treatment cannot be made, although all patients showed some response.

## 6. Conclusions

The concept of using diphtheria toxin in cancer treatment is not new but evolving. Alternative therapies to traditional chemotherapy are needed to improve outcomes, especially in patients with primary refractory or relapsing hematologic malignancies. Using IL-3 as a conjugate offers an advantage because multiple hematologic malignancies express CD123 and CDw131. Therefore, having a target that is highly expressed on tumor cells will increase the likelihood that the drug will exhibit anti-tumor activity by internalizing the diphtheria toxin and inducing apoptosis. The most striking activity was noted in BPDCN, with excellent responses in the affected organs, including bone marrow, skin, and lymph nodes. Outcomes in other hematologic malignancies, such as multiple myeloma and chronic myelomonocytic leukemia, are still in early clinical trials, and these studies are providing a better understanding of the bone marrow microenvironment and the role of pDC in tumor progression and treatment resistance. It has been reported that pDCs have anti-tumor activity through the production of interferon alfa [32].

## Figures and Tables

**Table 1:** Summary of SL-401 clinical trials in various hematologic neoplasms, baseline characteristics, adverse events and clinical outcomes (See page 14). Abbreviations: NCT, National Clinical Trial; R/R, Relapsed or refractory; AML, Acute myeloid leukemia; MDS, Myelodysplastic syndrome; CMML, Chronic myelomonocytic leukemia; BPDCN, Blastic plasmacytoid dendritic cell neoplasm; MM, Multiple myeloma; ORR, Overall response rate; CR, Complete response; PR, Partial response; LFT, Liver function tests; ASCO, American Society of Clinical Oncology; ASH, American Society of Hematology; CLS, Capillary leak syndrome; NA, Not available.

SL-401 Clinical Trial Registry Numbers	Primary Malignancy	Status	Study Start Date	Phase	Published Data	Number of Patients	Number of Cycles	Age	Male/Female	Adverse Cytogenetics	Intermediate Cytogenetics	Relapsed (any) and Refractory	Adverse Events	ORR	CR	PR
NA	R/R or elderly AML and high risk MDS	Completed	NA	I	Leukemia and Lymphoma 2007	45	1	67 (32–81)	23/22	17	25	35	Grade III LFTs, Grade II fever, chills, low albumin and hypotension	NA	1	3
NCT00397579	BPDCN	Completed	May 2013	I/II	Blood 2014 for the BPDCN	11	1	70 (40–77)	11/0	NA	NA	7	Grade IV thrombocytopenia, Grade III LFTs and neutropenia	NA	5	0
NA	RR/AML and BPDCN	NA	NA	NA	ASH 2015	17	multiple	63	NA	NA	NA	NA	Grade V CLS, Grade IV CLS, Grade III LFTs	NA	4	NA
NCT02270463	Consolidation Rx in adverse risk AML CR1	Recruiting	February 2015	I/II	NA	NA	NA	NA	NA	NA	NA	NA	NA	NA	NA	NA
NCT02268253	Advanced high risk MNP (SM, PED, MF, CMML)	Recruiting	December 2014	I/II	ASH 2016	19	multiple	69 (42–81)	NA	NA	NA	19	Grade III thrombocytopenia and anemia	NA	1	NA
NCT03113643	With AZA for Rx naïve AML/high risk MDS not eligible for standard Rx	Recruiting	June 2017	I	NA	NA	NA	NA	NA	NA	NA	NA	NA	NA	NA	NA
NCT02661022	R/R MM	Recruiting	January 2016	I/II	ASH 2016	2	multiple	65 (63–67)	NA	NA	NA	2	Grade II thrombocytopenia and hypoalbuminemia	NA	0	2
NCT02113982	BPDCN and R/R AML	Recruiting	September 2014	I/II	ASCO 2016 (only BPDCN)	18	multiple	70 (45–82)	NA	NA	NA	10	CLS and thrombocytopenia	87%	8	NA
